# The effect of a suspension training on physical fitness, lower extremity biomechanical factors, and occupational health in Navy personnel: a randomized controlled trial

**DOI:** 10.1038/s41598-024-61933-3

**Published:** 2024-05-16

**Authors:** Esmaeil Mozafaripour, Hossein Shirvani, Sobhan Alikhani, Mohammad Bayattork, Zohreh Yaghoubitajani, Lars Louis Andersen

**Affiliations:** 1 Health and Sports Medicine Department, Faculty of Physical Education and Sport Sciences, University of Tehran, between 15th and 16th St., North Kargar st., Tehran, Iran; 2 Exercise Physiology Research Center Research Institute for Life Style Baqiyatallah University of Medical Sciences, Tehran, Iran; 3 Faculty of Sport Sciences, Shahid Rajaee Teacher Training University, Tehran, Iran; 4 Sport Sciences and Physical Education, Faculty of Humanities Science, University of Hormozgan, Bandar Abbas, Iran; 5 Department of Health and Sport Rehabilitation, Faculty of Sport Sciences and Health, Shahid Beheshti University, Tehran, Iran; 6 National Research Centre for the Working Environment, Copenhagen, Denmark; 7 Department of Health Science and Technology, Aalborg University, 9220 Aalborg, Denmark

**Keywords:** TRX, Military, Physical performance, Quality of life, Health care, Health occupations, Risk factors

## Abstract

Optimal physical fitness is essential for military personnel to effectively meet their rigorous physical demands. This study aimed to investigate the effectiveness of a suspension training program on physical fitness, biomechanical risk factors for lower extremity injury, mental health, and work-related factors in Navy personnel. A total of 50 young men participated in a randomized controlled trial. The participants were randomly assigned to two groups (n = 25): the intervention group and the control group. The intervention group performed an eight-week suspension training session three times per week, while the control group maintained their daily duties. The primary outcome was physical performance. The secondary outcomes were determined biomechanical risk factors for lower extremity injuries, mental health, and work-related factors. The data were analyzed using the analysis of covariance (ANCOVA). Compared with the control group, the intervention group showed significant improvements in physical performance, biomechanical risk for lower extremity injuries, and work-related factors from baseline to follow-up (p ≤ 0.05). However, there was no improvement in mental health. Based on these findings, suspension training positively impacted physical fitness, reduced injury risk, and enhanced the work-related factors of Navy personnel. This study provides new insights for various related experts and military coaches because it is an easy-to-use and feasible method with minimal facilities.

## Introduction

Military personnel require optimal fitness to fulfill the physical demands of their occupational and combat-specific tasks^1^. Therefore, applying appropriate physical training programs is crucial for survival and combat success in this population^2^. Traditional training methods in military forces have predominantly emphasized aerobic endurance exercises, such as long-distance running and calisthenics. This approach has been favored due to its ease of prescription for training large numbers of soldiers^2^. However, functional muscle strength is crucial for meeting military demands, a need that cannot be fully addressed by aerobic exercise alone, both during training and under combat conditions^3^.

In addition, physical training can lead to acute and long-term overuse injuries^3^. For example, routine training, such as long-distance running, is a primary risk factor for foot injuries among military personnel^4–6^. It is hypothesized the overuse injuries such as stress fracture are one of the most common type of injury in military personnel^3^. The standard recommended strategy for injury prevention is to reduce training volumes^5–7^. Thus, to maintain and improve military personnel's physical fitness, alternative and safe training is necessary to be considered in some locations, such as those serving warships, submarines or limited military camps, where there is no possibility of performing exercise, such as running.

Further, other kinematic factors known as faulty movement patterns may increase the risk of lower extremity injuries, including knee valgus, excessive hip adduction and internal rotation, medial knee displacement, or a combination of these^8–10^. Furthermore, performing combat-specific training, including jumping, stopping, starting, bounding, climbing, pushing, and sprinting in conditions of poor neuromuscular control, may put military personnel at risk of lower extremity injuries^11^. Based on previous research, lower extremity faulty movement patterns and kinematics can be modified by applying injury prevention programs. As such, a combination of balance, strengthening, stabilization training, plyometric, or agility exercises can be applied in civilian athletes and nonathletic individuals^12–14^. Nevertheless, the effects of suspension training, which involves most of the mentioned factors required to improve lower extremity kinematics among military personnel, are unclear.

Moreover, warfare exposes military personnel to a high risk of potential traumatic events, causing stressful conditions associated with various mental health disorders and affecting their quality of life and job performance^15,16^. While physical activity improves health-related quality of life and productivity in healthy individuals, its effects on mental health and job performance in navy personnel are unknown^17,18^.

Suspension training, as a feasible and safe method, is presently applied in various fields since all physical fitness components, including flexibility, cardiovascular function, power, muscular strength, and endurance, can be simultaneously applied in a small space^19^. Total resistance exercise (TRX) training is a form of suspension training that uses body weight for resistance, focusing on strength, balance, flexibility, and core stability. It is adaptable for all fitness levels, supports a variety of exercises, and is portable for convenience. Studies suggest TRX as effective as traditional weight training for physical fitness^20^.

Consequently, such a form of training would be beneficial in the Navy, where personnel are mostly stationed in limited settings such as battleships and submarines. Additionally, previous studies have suggested that suspension training can enhance performance and motor control and that exercising in unstable conditions may reduce the incidence of injuries^21–23^. In this vein, no prior studies have investigated the efficacy of a suspension training program for improving performance, decreasing the risk factors for injuries, or increasing mental health in military personnel.

The present study evaluated the effectiveness of an eight-week suspension training program on physical fitness, lower extremity kinematics, mental health, and job performance among Iranian Navy personnel. We hypothesized that suspension training would improve physical fitness, lower extremity kinematics, mental health, and work-related factors in this population.

## Results

Among the 50 participants originally assigned to two groups, 42 individuals (n = 21/group) completed the study process (4 participants dropped out of each group: 5 for losing the posttest assessment and 3 for uncompleted intervention protocol), and the related data were included in the data analysis. No significant differences were found in the baseline demographic characteristics or dependent variables (P > 0.05) Table 1.

**Table 1:** Demographic information of subjects.

Variable	Intervention group	Control group	T value	P value
Age (year)	26.5 ± 2.5	26.4 ± 1.5	0.907	0.81
Height (centimeter)	178.9 ± 7.2	179.8 ± 3.5	0.225	0.44
Weight (kg)	76.7 ± 2.5	74.7 ± 3.9	0.932	0.58
BMI	24.8	24.78	0.990	0.31

Table 2 displays the ANOCVA results revealing significant differences after eight weeks of suspension training. In addition, the physical fitness tests indicated significant improvements in Cooper’s 12-min run test (F = 37.46, p = 0.001, η^2^ = 0.77), push-up (F = 37.20, p = 0.001, η^2^ = 0.48), and sit-up in 60 s (F = 60.68, p = 0.001, η^2^ = 0.60) variables in the intervention group in comparison to the control group during follow-up. Furthermore, no significant differences were detected in the deep squat jump test at follow-up (F = 10.25, p = 0.73; η^2^ = 0.004).

**Table 2:** Result of ANCOVA test.

Variable	Intervention group		Within-group mean differences	ANCOVA			Control group		Within-group mean differences	Between-group mean differences	%95 CI	
	Pretest	Posttest		F	P	η^2^	Pretest	Posttest				
											Lower	Upper
Cooper’s test (meter)	1811.90 ± 318.94	2066.66 ± 289.10	− 254.76	34.46	0.001*	0.77	1757.61 ± 233.98	1788.09 ± 219.03	30.48	278.57	127	442.5
Push-ups (number)	28.61 ± 9.72	34.42 ± 8.08	− 5.81	37.20	0.001*	0.48	26.38 ± 6.9	26.42 ± 5.58	1.09	8	3.1	12.9
Sit-ups (number)	32.85 ± 9.38	40.61 ± 7.27	− 7.76	60.68	0.001*	0.60	34.21 ± 7.12	33.09 ± 5.37	1.12	7.52	3.8	11.24
Deep squat jump (centimeter)	224.42 ± 19.13	225.71 ± 15.91	− 1.29	10.25	0.73	0.004	221.85 ± 13.01	224.61 ± 15.27	− 2.76	1.1	− 0.15	2.35
Knee varus/valgus (degree)	14.68 ± 2.01	12.00 ± 1.19	2.68	38.02	0.001*	0.44	14.32 ± 2.89	13.88 ± 2.42	− 1.88	− 1.88	− 3.84	0.08
Hip adduction/abduction (degree)	8.80 ± 1.63	6.72 ± 1.69	2.07	28.93	0.01*	0.38	8.04 ± 1.54	8.24 ± 2.04	− 1.5 2	− 1.52	− 2.2	− 0.84
Tibia adduction/abduction (degree)	7.04 ± 1.36	4.40 ± 0.91	2.64	165.32	0.001*	0.77	7.00 ± 1.29	7.24 ± 1.05	− 2.84	− 2.84	− 3.11	− 2.57
FMS (score)	14.00 ± 2.02	17.00 ± 1.48	− 3	52.96	0.001*	0.57	13.61 ± 1.39	13.80 ± 1.50	− 0.19	3.2	3.17	3.23
Work ability index (score)	6.24 ± 1.50	8.52 ± 0.82	− 2.28	87.58	0.01*	0.65	7.06 ± 1.87	5.60 ± 1.44	2.92	2.92	1.71	4.13
Job satisfaction (score)	6.88 ± 1.20	7.48 ± 0.87	− 0.6	12.51	0.01*	0.21	6.64 ± 1.31	6.56 ± 1.19	0.92	0.97	0.35	1.6
Mental health (score)	70.80 ± 6.15	70.36 ± 5.83	0.44	1.04	0.31	0.02	69.88 ± 7.04	68.44 ± 6.67	1.92	1.92	− 0.6	4.44

*Significant difference at level of *P* < 0.05.

In general, the results demonstrated significant changes in all kinematic variables in the intervention group compared to the control group at follow-up: knee valgus/varus (F = 38.02, p = 0.001, η^2^ = 0.44), hip adduction/abduction (F = 28.93, p = 0.01, η^2^ = 0.38), tibia adduction/abduction angle (F = 165.32, p = 0.001, η^2^ = 0.77), and FMS (F = 52.96, p = 0.001, η^2^ = 0.57).

Regarding work-related parameters, significant improvements were observed in the Work Ability Index (F = 87.58, p = 0.01, η^2^ = 0.65) and job satisfaction (F = 12.51, p = 0.01, η^2^ = 0.21). However, no significant differences were exhibited in mental health (F = 1.04, p = 0.31; η^2^ = 0.02).

## Discussion

The present study revealed significant improvements in physical performance, biomechanical risk factors for lower extremity injuries, and work-related parameters following 8 weeks of suspension training in military personnel.

Traditionally, military training has focused on aerobic endurance exercises, particularly long-distance running, due to its feasibility for large-scale training of soldiers. However, such exercises may not fully meet the diverse demands of military personnel. Importantly, in confined environments such as warships or submarines, the feasibility of traditional aerobic exercise is further limited. Additionally, these methods might increase injury risk^4–6^, particularly in the context of poor neuromuscular control and uncontrolled movements^7^.

Given the frequent location changes and unique challenges faced by military personnel, adaptable and easily implementable training methods are needed. Suspension training, which effectively enhances various aspects of physical fitness, including flexibility, cardiovascular fitness, power, muscular strength, and endurance, is a suitable alternative^12^. This modality offers a comprehensive approach to physical conditioning, addressing the multifaceted requirements of military fitness.

Suspension training positively affects physical performance in military personnel, probably due to the repetition training continuum theory^24^. This theory mainly concerns the type of adaptation caused by the number of repetitions considering resistance in the human body. In addition, based on this theory, exercises with high intensity and low repetition, including strength, improve factors, and those with low intensity and high repetition with short rest between sets (implemented in our study) improve muscular endurance^25^. Furthermore, the oxidative capacity of skeletal muscles can progress through high-intensity exercise^26^. Similarly, it likely contributes to reducing reliance on energy production from the substrate-level phosphorylation pathway to decrease the production and accumulation of metabolic substances such as lactate in the muscle and blood, which ultimately causes muscle fatigue delay^27^. Therefore, training intensity improved muscle endurance through the application of the current intervention and through the use of suspension exercises with TRX, emphasizing the use of body weight with high repetition and lack of rest time within a circuit. Such a result was evident as the main issue in some tests, such as push-up and sit-up, leading to improved scores. Additionally, the present results indicated an improvement in running test scores, indicating that aerobic capacity improved in the intervention group.

As a plausible explanation for the present improvement in performance resulting from suspension training, high-intensity circuit exercises affect various molecular signaling pathways, such as Ca2 + /calmodulin-dependent protein kinase, AMP-activated protein kinase, Sirtuin 1 and peroxisome proliferator-activated receptor gamma coactivator 1-alpha^28–31^. All of these pathways can improve the oxidative capacity of skeletal muscle, delay fatigue, and subsequently increase the performance of endurance activities such as endurance running. In addition, the increase in the capacity of the cardiovascular system results in oxygen delivery after these exercises are implemented. Thus, through circuit exercises, the upper and lower body muscles simultaneously force the heart to contract more strongly to deliver oxygen to active muscles^32^. Therefore, heart muscle strength and stroke volume improve to maximum cardiac output, resulting in easier delivery of oxygen to active muscles and discharge of metabolic substances, resulting in progressing endurance performance during tests such as running^33^.

Moreover, the results revealed significant improvements in the quantitative and qualitative risk factors for biomechanical lower extremity injury in the intervention group. Previous studies have reported that exercising under unstable conditions can stimulate the sensorimotor system, providing the necessary response to maintain postural stability and improving movement accuracy^34,35^. A lack of proper stability during exercise, as applied in the present study, changed the length of the muscle–tendon unit and neuromuscular activity, immediately leading to challenges in efferent activity to achieve a more accurate movement pattern^36,37^. Additionally, closed-chain exercises involving multijoint movements under unstable situations integrate the data used for postural adjustment^35^. In addition, training under unstable conditions improves muscle cocontraction^36^, progressing motor control and movement integration^36^.

Considering the above, the rationale of developing suspension exercises with TRX is to create an unstable surface to stimulate neuromuscular parameters and enhance central nervous system control over the movements of the musculoskeletal system^20^. In this vein, the exercise programme used in this research, considering the neuromuscular mechanism, likely improved the biomechanical factor in the intervention group.

Based on the present results, work ability and job satisfaction differed significantly between the intervention groups. This finding is in line with the literature indicating positive effects of physical fitness on work ability and job satisfaction^38–40^. As such, individuals with higher levels of physical fitness and better cardiovascular efficiency experience less fatigue, have better focus on their jobs and are more satisfied with their quality of work^40^. Accordingly, all the physical fitness components improved through the intervention applied in this study among military personnel. Therefore, such results may lessen physical fatigue, leading to progress in work ability and job satisfaction. The lack of significant change in mental health among military personnel post-intervention, despite improvements in work ability and job satisfaction, could be attributed to several factors. These include the possibility that the measurement tools lacked sensitivity, or individual responses varied greatly. Additionally, pre-existing mental health conditions, stigma around reporting such issues, and external stressors could have also played a role in the unchanged mental health outcomes. It's crucial to consider these elements when evaluating the intervention's effectiveness and planning subsequent measures.

A limitation of this study is its exclusive focus on male personnel within a narrow age range. Additionally, the inability to control for daily activities and the potential impact of military duties on the results limits the generalizability of our findings to the broader military population. Furthermore, unlike many interventional trials, a double-blind design was not feasible in this study, which may affect the interpretability and applicability of the results.

## Conclusion

The current study revealed that 8 weeks of suspension training was effective for Navy personnel. Based on the present findings, using this method increased physical fitness levels and decreased the biomechanical risk factors for lower extremity injuries. This approach likewise improved the work-related parameters of this population. In addition, due to the easy accessibility, feasibility, safety, and efficacy of such training being performed with limited equipment and space, it is recommended that it can be applied by coaches and military supervisors because of the specific conditions of Navy personnel.

## Methods

### Study design

This parallel-group RCT studied a suspension training program in which 50 male participants (18 to 28 years old) were randomly assigned to two groups: the intervention group and the control group. The intervention group performed an eight-week suspension training program plus their daily routines, and the control group continued their daily military activities. Baseline and follow-up assessments were performed after eight weeks of intervention. The follow up assessment were performed after at least 48 h after last training session.

**Figure 1:**
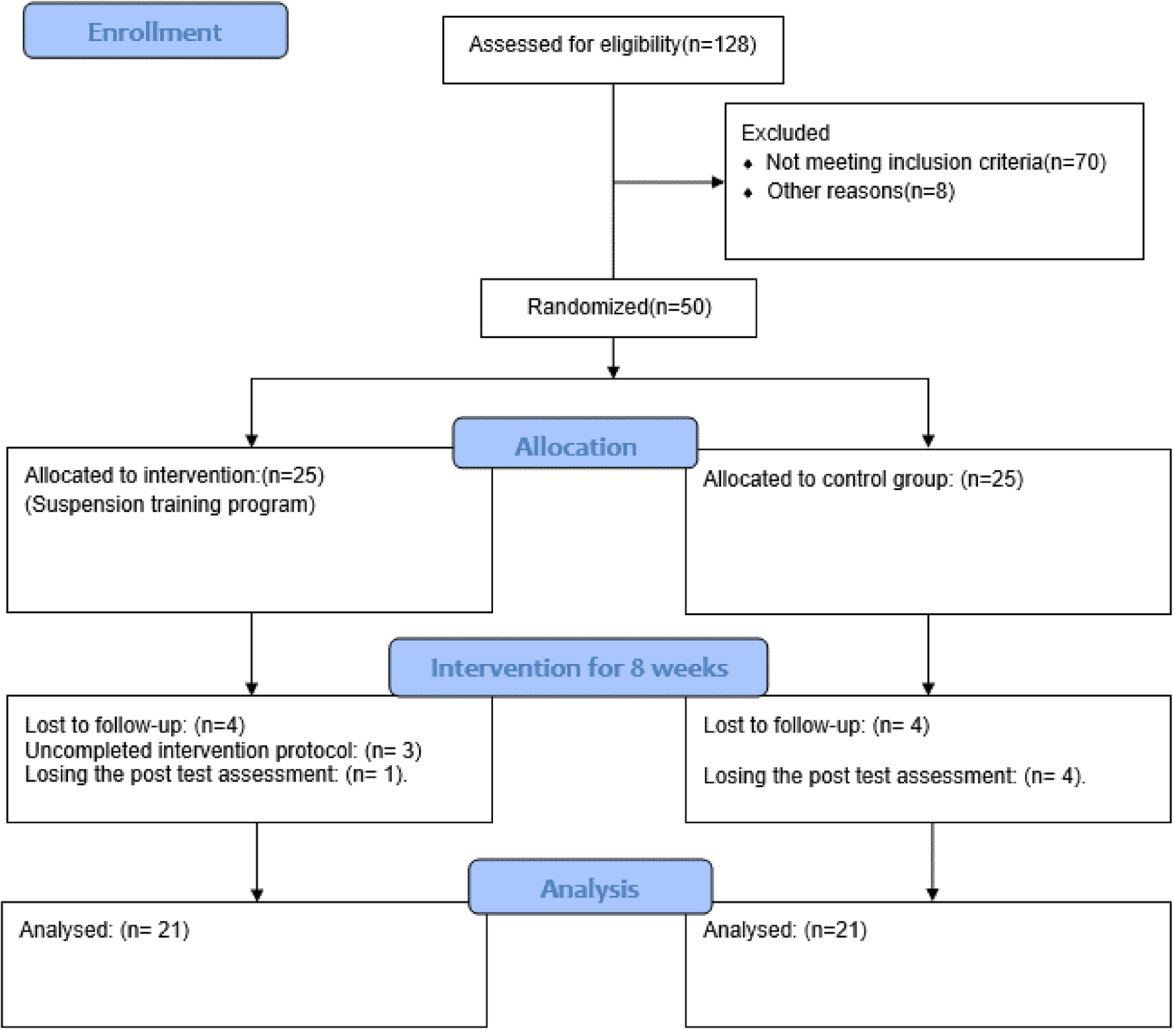
Consort flow diagram.

The trial was conducted following the CONSORT guidelines and the study consort flow diagram is presented in Fig. 1. At the commencement of the study, the demographic data of all participants were collected, and an informed consent form was signed by each participant. Ethics approval was issued by the Ethics Committee on Research at Baqiyatallah Hospital, Iran (IR.BMSU.BAQ.REC.1398.016/ 28/01/2020). The experimental conditions adhered to the principles outlined in the Declaration of Helsinki. The protocol of the current study was approved by the Iranian Registry of Clinical Trials on 13/06/2020 (IRCT20180821040843N2). The data of all participants were secured in a locked computer system in areas with limited access.

### Study participants and eligibility criteria

G*Power software (version 3.1.9.2; Kiel, Germany) was used to estimate the optimal study sample size. A total of 20 participants were included in each group to determine the statistical significance at 80% power, with d = 0.70 indicating an effect size. Considering a dropout rate of approximately 20%, 25 participants were recruited and allocated to each group from the Iranian Navy personnel serving in the Bandar Abbas military base. The participants were excluded from the study with a history of lower extremity injury making movement restriction^41,42^, the existence of any visible musculoskeletal deformities in the lower and upper extremity in normal standing posture^43,44^, not attending the training sessions more than three times or two sequential sessions, having abnormal body mass index (BMI) less than 18 and more than 28^45^. All the participants were allowed to leave the study at any time.

### Randomization

Computer-generated block randomization in a 1:1 ratio was applied for randomization followed by a concealed allocation through opening sequentially numbered, checkmate, and sealed envelopes. As such, a card inside indicated that the participant was randomly allocated to either the intervention or control group. To address missing data, a particular procedure was performed at a data review meeting before starting the statistical analysis. The data analysts and outcome assessors were blinded to the participants’ groups.

### Intervention

Circuit resistance training is one of the best methods for improving cardiovascular fitness^33,46,47^. In addition, suspension training performed under unstable conditions may affect performance and motor control, positively contributing to a decrease in the incidence of injury^21–23^.

The intervention in this study was designed for circuit resistance workouts using suspension training with the TRX band to address all physical fitness demands required by military personnel. The training was performed in the shortest amount of time with the minimum number of facilities needed to accomplish the occupational and combat-related specific tasks.

An eight-week three-time per week suspension training program was prescribed using the TRX band in three phases (initial, improvement, and maintenance) based on the circuit design. The participants performed the first exercise in the circuit with a predetermined number of repetitions and continued the next exercise with no rest time until all the exercises in the circuit were completed and the circle was ended.

**Figure 2:**
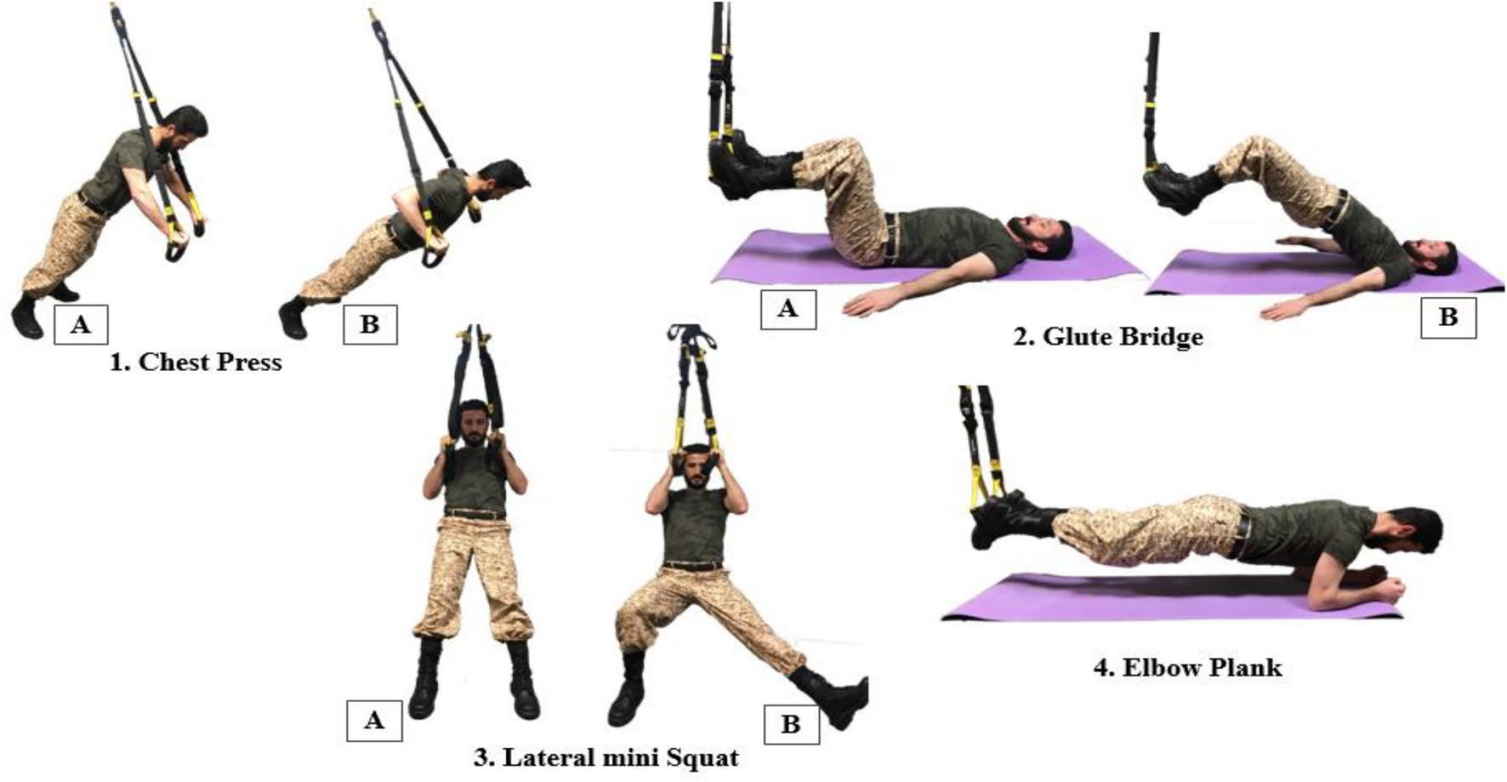
Exercises 1–4 (initial phase).

The initial phase (anatomical adaptation) was implemented within weeks 1–2 (exercises 1–4). This phase was performed in circuit form to provide anatomical adaptation to transfer the participant to the subsequent phases (Fig. 2). Therefore, the participants’ optimal concentration was needed because this stage emphasized cognition, purposing the quality and learning of the exercises^33,20,48^. Each session took approximately 30 min to complete.

**Figure 3:**
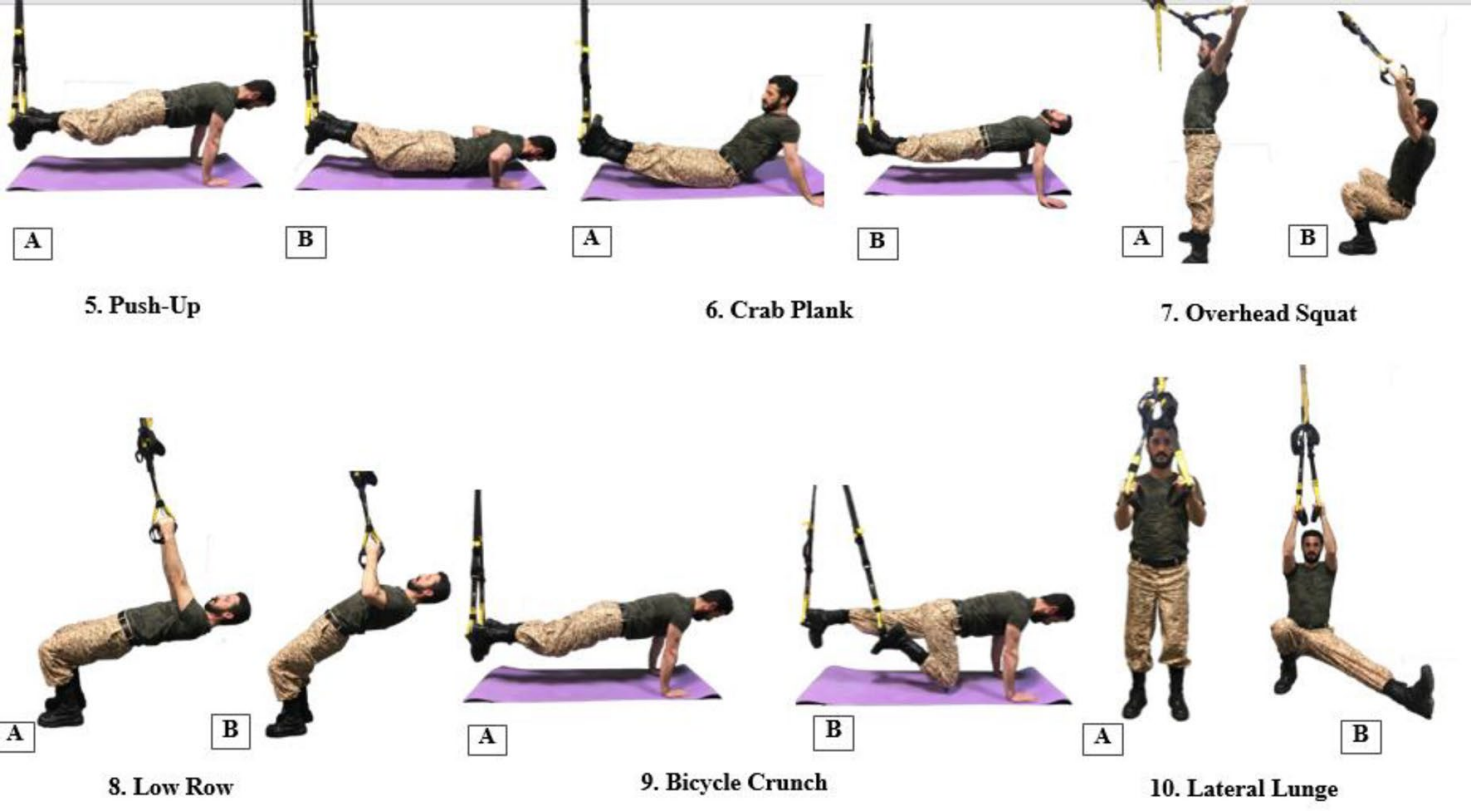
Number of exercises 5–10 in weeks 3–4 of the improvement phase.

**Figure 4:**
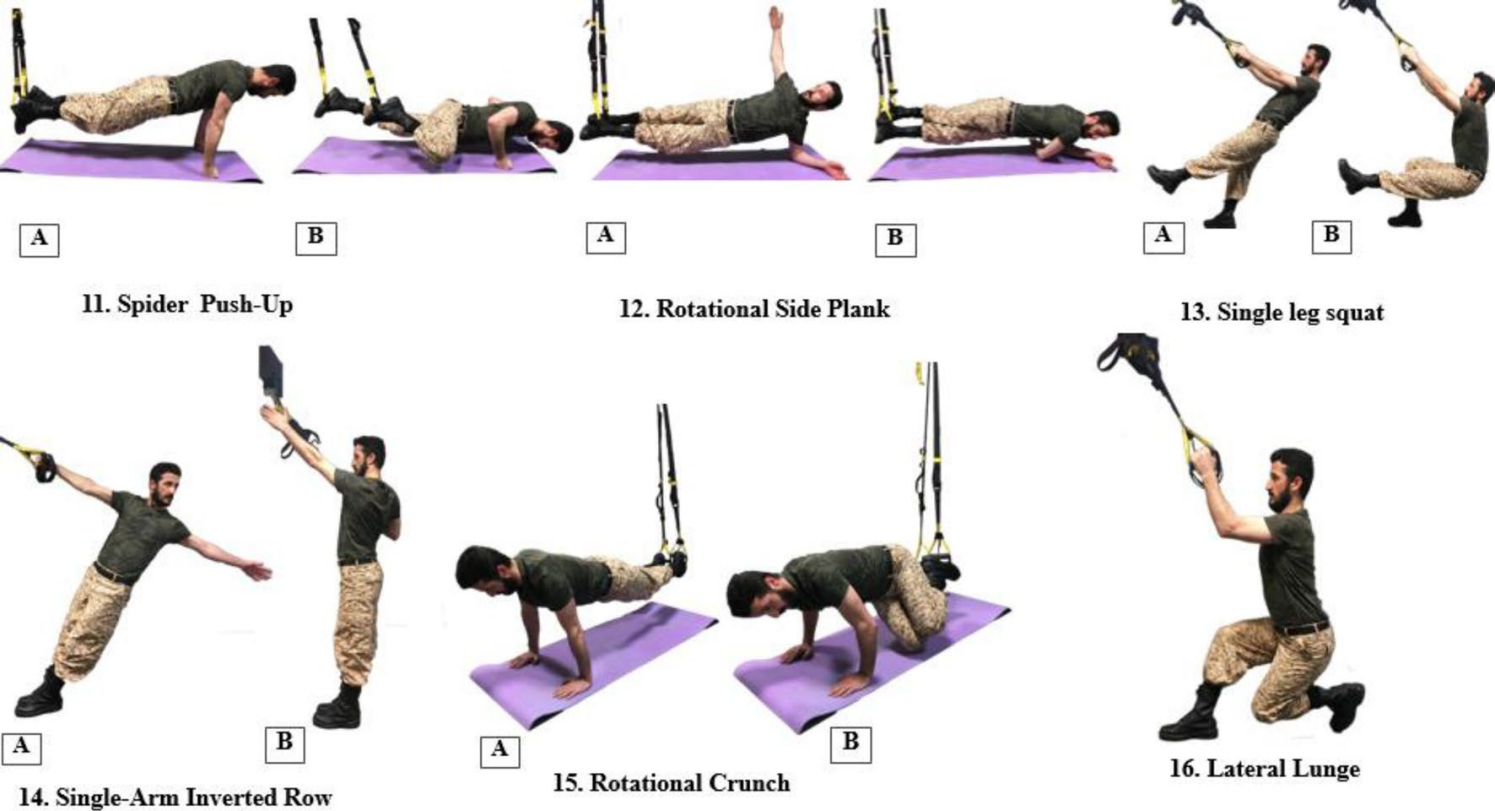
Number of exercises 11–16 in weeks 5–6 of the improvement phase.

The second phase (improvement) was implemented within weeks 3–6 (exercises 5–16) and aimed to create the necessary tissue adaptations as the main part of the training program in which most progress occurred^49^. To achieve optimal adaptation, training progress should include the number of exercises, circles, repetitions, and additional demanding exercise (Figs. 3, 4)^46^. Each session took approximately 45 min to complete.

**Figure 5:**
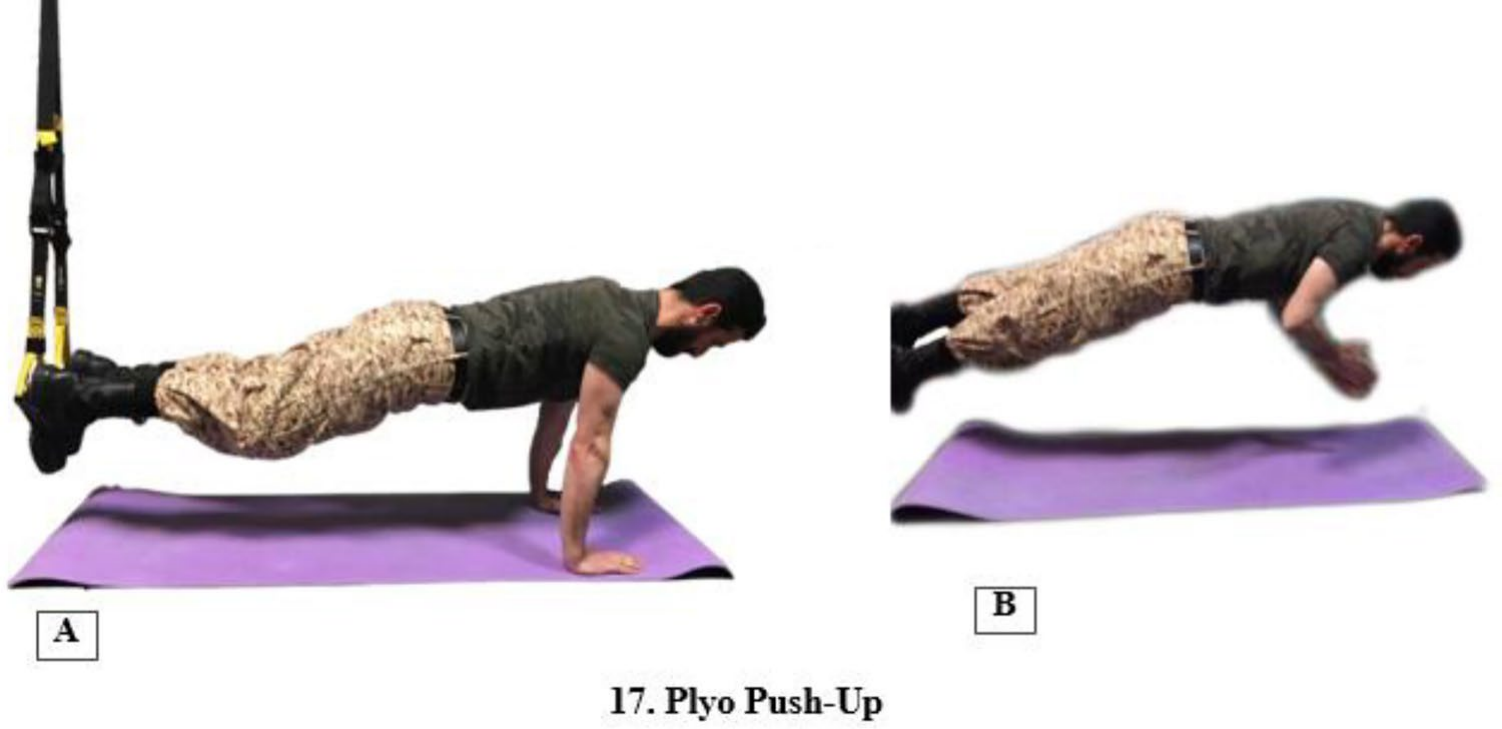
Exercise number 17 in the maintenance phase.

The third phase (maintenance) was completed during weeks 7–8, during which the patients purposed to stabilize the first two phases and provide a maximum challenge for the neuromuscular system to achieve the best neuromuscular adaptation^48,49^. In this stage, one exercise (17) was performed along with all of the exercises in the previous two phases (Fig. 5). The detailed intervention programs can be found in the article published by Shirvani et al.^50^. Each session took approximately 50 min to complete in this phase.

### Study outcome measures

The main researcher measured all variables of interest at baseline and at follow-up after 8 weeks, and physical fitness levels were included as the primary outcome. The risk factors for injury to the biomechanical lower extremity, mental health, and work-related parameters were considered secondary outcomes.

### Study procedure

To assess physical fitness levels, the following tests were applied and found to be strongly correlated with physical performance among military personnel^51^.

- Cooper’s 12-min run: for cardiovascular function;
- Deep squat jump: for lower extremity muscle strength;
- Push-up in 60 s: for upper extremity muscle strength and endurance;
- Sit-up in 60 s: for core muscle strength and endurance^51^.

### Lower extremity biomechanical factor

Dartfish™ Pro Suite Software was used to determine the risk factors for injuries in the frontal plane kinematic data collected from the biomechanical lower extremity. The required data were collected during the drop-jump task to evaluate the following variables: knee valgus/varus and hip adduction/abduction angles and tibia adduction/abduction angles. To collect the required data, 12 reflective markers were placed on anatomical landmarks as follows: the ASISs, lateral side of tights, lateral epicondyles of the knee, lateral side of shanks, lateral malleolus, and center of the calcaneus. Then, the participants were requested to perform a drop jump sequence by jumping off the box at 40 cm height, landing, and immediately performing a maximum vertical jump^52^. Each participant was requested to land precisely in front of the box to be in the correct position for the camera to record properly, with no instructions for the performance procedure. A digital video camera was used to record the data, which were placed three meters away from the greater trochanter's height to be aligned perpendicularly to the participant’s frontal plane^53^. Biomechanical data values were recorded at their maximum value at the landing phase. The average of three trials of the recorded videos was transferred to DartFish software for kinematic variable analysis.

Furthermore, a trained assessor administered the functional movement screen (FMS) as an additional tool containing seven tests scored on an ordinal scale with four categories. Then, seven fundamental movement pattern tests were conducted to calculate the biomechanics that are quantitatively related to athletic movements, including deep squats, inline lunges, hurdle steps, shoulder mobility, active straight leg raises, push-ups, and rotary stability^54,55^.

Each participant performed the tests three times, and a score ranging from 0 to 3 was recorded (3: the best, 0: the worst)^54^. Finally, all the processes were recorded by a digital camera and observed by the main examiner visually for further action.

### Mental health and work-related parameters

The 36-item short form health survey (SF-36) was administered as a self-administered questionnaire containing 36 items to measure the health perceptions of military personnel^56^. It took approximately 5 min to complete and measure overall health on eight multi-item scales covering functional status, well-being, and comprehensive health evaluation. In addition, such a questionnaire has been widely applied among military personnel in previous studies^57–59^. In the present study, we used the Iranian-translated version of the SF-36 questionnaire, which has appropriate validity^60^. Furthermore, we included a single-item question from the Work Ability Index and job satisfaction questionnaires. Participants rated their present work ability and job satisfaction related to their lifetime best work experience on a scale of 0–10^61^.

### Statistical analyses

IBM SPSS version 20 for Windows (SPSS, Inc., Chicago, IL, USA) was used to analyze the collected data statistically. The Shapiro–Wilk test was applied to analyze the statistical distribution and variance homogeneity. Using ANCOVA, the different scores were displayed from baseline to follow-up as the outcome measure (dependent variable), and the independent variable was the group (control vs. intervention). In addition, the analysis was controlled for the baseline values; thus, in the case of significant differences at baseline between groups for the descriptive variables, such data were applied as control variables in a sensitivity analysis. Descriptive values (means ± SD) for each variable and the effect sizes calculated via partial eta-squared (η^2^) tests. Effect sizes were interpreted as small (0.01), medium (0.09), or large (0.14)^62^. The alpha level in this study was set at α = 0.05.

## Data Availability

The datasets generated during and/or analysed during the current study are available from the corresponding author on reasonable request.
